# The Impact of a Researcher’s Structural Position on Scientific Performance: An Empirical Analysis

**DOI:** 10.1371/journal.pone.0161281

**Published:** 2016-08-31

**Authors:** Damien Contandriopoulos, Arnaud Duhoux, Catherine Larouche, Mélanie Perroux

**Affiliations:** 1 University of Montreal, Faculty of Nursing, Montreal, Quebec, Canada; 2 University of Montreal Public Health Research Institute (IRSPUM), Montreal, Quebec, Canada; 3 Research Centre–Charles-Le Moyne Hospital, Longueuil, Quebec, Canada; 4 McGill University, Department of Anthropology, Montreal, Quebec, Canada; Universite Toulouse 1 Capitole, FRANCE

## Abstract

This article discusses the nature and structure of scientific collaboration as well as the association between academic collaboration networks and scientific productivity. Based on empirical data gathered from the CVs of 73 researchers affiliated with an academic research network in Canada, this study used social network analysis (SNA) to examine the association between researchers’ structural position in the network and their scientific performance. With reference to Granovetter’s and Burt’s theories on weak ties and structural holes, we argue it is the bridging position a researcher holds in a scientific network that matters most to improve scientific performance. The results of correlation scores between network centrality and two different indicators of scientific performance indicate there is a robust association between researchers’ structural position in collaboration networks and their scientific performance. We believe this finding, and the method we have developed, could have implications for the way research networks are managed and researchers are supported.

## Introduction

The structure, nature and strength of social connections with peers, partners and friends deeply influence numerous aspects of human life. From how people get jobs, to market success or organizational conflicts, people’s positions in social networks matter [1–6]. Similarly, in the field of academic research, the creation of research centres or networks stems from the idea that collaboration ties will positively influence researchers’ performance by enabling the exchange of resources, knowledge and experience [7]. Funding agencies and universities are now particularly involved in supporting the formation of institutional networks [8]. The question then is whether these structures aimed at supporting and strengthening collaborative research actually improve scientific achievements. One challenge is that researchers' research networks are highly overlapping structures, making it difficult to assess the impact of any one formal research structure on a researcher’s performance [9]. Given the strong competition to secure research funding and performance-based allocation methods, finding a way to improve the effectiveness of such institutional networks can have important implications.

The present paper has three objectives. The first is to discuss the conceptualization of scientific collaboration networks beyond co-authorship and their role in scientific endeavour. The second is to present a scientific collaboration mapping method based on social network analysis (SNA) and illustrate it empirically using data collected on a provincially funded research network in Quebec (Canada). The third is to assess the association between researchers’ structural position in this network and two measures of scientific productivity and impact. We conclude by discussing the potential implications of these findings for creating more effective research networks.

## Conceptualizing Scientific Networks

The idea that networking and belonging to dynamic communities and research groups positively influence scientific productivity has strong face value for anyone working in the field. Researchers benefiting from peer support and the sharing of ideas, expertise and pooled resources provided by collaboration in peer networks are likely to have higher productivity rates than those more isolated. A number of studies have demonstrated the relation between collaboration and higher scientific performance [10–17]. Recent works tend to shift the emphasis from the comparison of overall outcomes (publications, funding received, etc.) of different research networks or university departments to the social structure of collaboration, with the use of SNA methods [16, 17].

However, few studies have analyzed the interplay between structures of collaboration, scientific productivity and institutional research networks. For example, Ponomariov and Boardman’s [8] longitudinal study of the publication records of researchers before and during their affiliation with a specific research centre suggested that the affiliation did enhance collaboration practices and publication productivity, mostly for junior faculty. Conversely, Ynalvez and Schrum showed that, in the context of resource constrained research institutions, it was informal professional network ties rather than affiliation with formal collaborative research groups that mattered the most for publication productivity [7]. Such analyses could be complemented by examining the kind of social structure of collaboration formed by the members of a specific research centre to better understand which collaboration ties influence scientific performance the most. The method presented in this paper is an attempt to achieve this goal.

First, it is important to note that the relevance of analyzing collaborative relations is rooted in the core conceptual question of research networks’ embeddedness within deeper social structures. It is beyond the scope of the present paper to discuss the interdependence of social structure and human behaviour, but the analysis proposed here posits that observable social connections are the products of unobservable underlying social structures [18]. The underlying social structures shaping one’s participation in scientific endeavour include both field-based socially structured rules, goals and values [19, 20] and individually defined social capital [2, 18, 19, 21]. In their simplest form, individual-level conceptualizations of social capital can be described as the sum of who you know, who knows you, and the trust built into those relationships. Social capital encompasses both actual contacts and potential contacts. Just as individual behaviour is driven by existing social structures while at the same time driving future social structures, actual inter-individual communications are an expression of current social capital and the structuring of future capital.

Our approach here is based on ideas very similar to those of Burt on the social structure of competition [2], in which social capital is seen as a personal asset that increases success in market-based competitions. Likewise, we argue that social capital has a direct influence on scientific production and researchers’ productivity. This is because network connections provide both an informational advantage (timely and accurate access to valuable information) and a control advantage (strategic positioning in relation to partners’ and competitors’ actions). With regard to causality, we posit that social capital drives actual work relations, which in turn influence academic productivity. In other words, there is the invisible network of connections driven by actualized and potential social capital, and there is the operationalization of this underlying structure into current work relationships. The method developed in this article uses an SNA approach based on actual work collaboration as a proxy for the underlying social capital structures of competition.

Attempting to measure the benefits of scientific networks and collaborations nevertheless presents significant conceptual and methodological challenges that have not been completely addressed by past studies. One of them concerns the types of network connection to be taken into consideration. Researchers will undoubtedly have direct interactions with one another, in person or through technological means, but the concept of a social network goes beyond regular or occasional direct contacts. For example, although direct contacts matter, a researcher might consider a collaborator on the other side of the globe with whom he or she has had no interaction for years as an ‘active’ contact, while considering the colleague with whom he or she just had a conversation in the corridor as not belonging to his or her network [22]. Collaborations established through co-authored publications have been the most common way of measuring academic work collaborations [13, 16, 17, 23–26]. Co-authored papers are a practical data source because they can be easily available and quantifiable. However, co-authorship networks only account for one specific form of collaboration, and many other forms of collaboration will not result in a co-authored paper [17, 27]. We aimed to sample inter-researcher connections as broadly as possible by including other possible forms of collaborations that remain easily available through academic CVs.

The network sample constitutes another important issue in studies of scientific networks. The simplest way to map a scientific network is to adopt an ego-centred view in which each researcher is, at any given time, in more or less regular and direct contact with colleagues, collaborators and acquaintances. To obtain meaningful results from SNA approaches we would need to analyze not only direct connections (a given researcher’s contacts), but also indirect connections (those contacts’ contacts) [1, 4, 6, 28, 29]. However, mapping large networks from recursive identification of contacts of contacts is an endless process. A more feasible approach is to aggregate ego-centred networks of direct connections for all egos in a defined group. This approach is easier to scale up and could in theory be extended to map scientific collaborations on a very large scale, such as for countries, disciplines, fields, or even the world.

For the sake of discussion, let us imagine the purely hypothetical scenario that there is, globally, at any given time, a finite and identifiable group of people involved in the human scientific endeavour. By aggregating the direct networks of all these people, we would obtain a reasonably accurate snapshot of the total ‘scientific community’. The point of this theoretical example is to recognize that any effort to map scientific collaborations on a smaller scale than this worldwide ‘community’ will necessarily involve focusing on more or less meaningful samples, or subsets, of that underlying community.

Finally, regarding network shape, casual observations of science at work suggest that scientific networks are complex networks characterized by small clusters of highly interconnected researchers embedded in the looser structure of the community. These groupings are often only very remotely anchored in geographic or institutional structures. They can be conceived as the result partly of idiosyncratic causes, but also, to some extent, of disciplinary and field structuring [20], institutional and national boundaries, methodological preferences, and the like. These clusters have taken many names in the scientific literature [30], such as invisible colleges [31], scientific communities [32], schools of thought, or social circles [33]. In such a heterogeneous network structure, with dense clusters connected through weaker links, Granovetter [1, 6] and Burt [2] suggest it is weak tie connections or one's position in a structural hole that will provide individuals with a structural advantage. Granovetter's and Burt's arguments are not entirely convergent and have been discussed at length elsewhere [34, 35], but both show convincingly that, structurally, competitive advantage stems not from strong and redundant links within clusters but rather from inter-cluster weak ties, and that it is those who are in such bridging positions who benefit the most. A few studies on collaboration and academic performance have presented results that can support these arguments. For example, both Ebadi and Schiffauerova’s [17] and Abbasi et al.’s [16] studies suggest there is a positive relation between a researcher’s bridging position in a network and its scientific performance. Ebadi and Schiffauerova add that, to secure the research funding necessary to increase scientific productivity, it is better to multiply and diversify relations rather than to maintain close ties with only a few prominent researchers.

This paper contributes to this literature not only by examining the links between collaboration ties and scientific performance, but also by examining how institutional networks intersect with actual structures of collaboration formed by researchers.

## Data Collection and Method

To develop our collaboration network analysis method and to assess the association between structural position and researchers’ productivity and influence, we used data from a Canadian provincial research network, hereinafter called the RN. The RN is a recently formed health-related disciplinary network funded since 2012 by the Fonds de recherche du Québec–Santé (FRQS) [Quebec's government health-research funding agency], the Ministry of Health and Social Services, and various university partners. The RN is one of 18 such provincial networks whose mandate is to strengthen research in their field and enhance national and international researchers' competitiveness. They aim to fulfill these objectives by strengthening inter-individual and inter-university collaboration as well as by improving the impact and visibility of their members’ research findings. The networks also provide both seed money for regular members’ applications to external funding competitions as well as financial support for students.

The RN includes members from eight different Canadian universities and was composed of 73 regular members at the time of data collection. Membership in the RN is voluntary, but members must fulfill a set of requirements pertaining to their research focus and academic position. By identifying the collaborators of each of the RN members, we obtained a larger social network of 2,360 researchers (73 RN members and 2,287 connected non-RN members) upon which we based our analysis.

### Measuring structural position

Measuring each researcher’s structural position in the RN involved three steps: identifying each researcher’s collaborations, aggregating individual collaboration networks into a single unified collaboration network, and then computing the structural position of each researcher.

In the first step, we identified ongoing collaborations using the latest available (2012) complete Canadian Common Curriculum Vitae (CCV) of each member of the RN. The CCV is a standardized web-based CV platform onto which all researchers can upload their CV information in a common format [36]. The CCV is now required by most federal and provincial public research funding organizations in Canada. To build the ego-centred collaboration networks, we compiled the names of other researchers appearing in each RN member’s CCV. As we aimed to sample inter-researcher connections as widely as possible, we included not only co-authors, but also collaborators recorded in the CCVs for ongoing grants and co-presented communications, as well as master’s, doctoral and postdoctoral students currently supervised or co-supervised. We considered as co-authors the collaborators named in published and accepted papers in peer-reviewed journals, book chapters and research reports. Given the variability of authorship practices, we considered all eligible publications regardless of authorship position. We also assigned a temporal length of collaboration to each type of research activity considered, to develop a reliable representation of each RN member’s network of collaborators at the time of their latest CCV. The choice of length of time to attribute to previous collaborations was subjective. We used one year prior to the actual date for a communication, two years before publication for an article, book, chapter or report, four years for a master’s student supervision, and six years for a doctoral student supervision.

In the second step, the 73 lists of collaborators compiled—one for each RN member—were aggregated into one unified network data set by transposing all the data collected into a single-mode matrix (collaborator X collaborator). Such a matrix is readily importable into SNA software [37, 38].

Finally, the third step was to analyze the data set from an SNA perspective. SNA is a trans-disciplinary approach focused on understanding the structure of relations connecting different elements. It has commonly been used to study the formation of informal communities or subgroups within institutional settings [38–40]. Scientific communities are an example of these types of networks. Interestingly for this research, SNA is also increasingly being used to understand the impact of networks on productivity [11, 41, 42].

We used the open source software Cytoscape 3.2.1 to analyze our data set. The analysis was based on two complementary methods. The first consisted of creating visual representations of the aggregated RN collaboration network. SNA data can be visualized as graphs, called sociograms, without information loss. In the sociograms we produced, a node represents a researcher and each collaboration tie is drawn as a line between two researchers. Using sophisticated algorithms derived from graph theory, sociograms can be optimized for visual analysis. We used the *Prefuse force directed layout* to optimize the visual representation of the sociogram and facilitate its analysis. This algorithm belongs to a particular approach called “force-directed” [43, 44]. Force-directed sociograms are optimized from the principle that nodes are mutually repulsive to each other, while ties constitute attractive forces. Force-directed algorithms ensure that repulsive and attractive forces are balanced so that interconnected nodes are closer to each other. In resulting sociograms, highly interconnected nodes will be pushed to the centre of the network and clusters of interconnected nodes (cliques) will be visible. We also plotted the graph such that node shape represents RN membership (members as round dots, non-members as lozenges), node size corresponds to degree (higher degrees correspond to bigger dots), and node colour represents betweenness centrality (continuous mapping: light green = smallest centrality and red = highest centrality). Degree and betweenness centrality (BC) are node-level structural metrics, which we present below.

The second method we used was a statistical analysis of this large network formed by the aggregation of each RN member’s personal collaboration networks. SNA software can be used to compute networks and calculate node-based metrics. We used two node-based metrics for the analysis: degree and BC. Degree is the simplest structural metric. It represents the number of collaborations (ties) linked to a researcher (node). In our mapping of the network, each tie corresponds to authorship of a paper or book, graduate student supervision, a communication, etc. Hence, in this study, degree is a rough measure of individual productivity. This direct correlation between degree and productivity, however, precludes using this metric to measure the association between structural position and productivity.

Whereas degree attributes the same value to any tie, BC values ties differently depending on their contribution to a node’s connectivity in the network. BC is computed as the proportion of all shortest paths in a network that pass through a given node [29, 38, 45]. As such, although it belongs to the many measures of network centrality [29, 46, 47], BC is quite independent from the notion of being close to the theoretical ‘centre’ of a network; rather, it has more to do with the notion of being an important, and thus central, bridge in a network.

### Measuring productivity and influence

There are multiple understandings of research performance and as many ways to measure it. In this study, the RN researchers’ performance was measured with two complementary indicators, one focused mostly on productivity and the other on influence. We believe productivity and influence represent overlapping but distinct dimensions of scientific performance.

The influence-focused performance metric we used was the h-index. The h-index (also called Hirsh index) is a bibliometric indicator that measures a scholar’s cumulative impact by combining the number of publications and their citation impact [see 48 on the calculation of the h-index]. Being one of the first indicators to combine both the volume and impact of individual publications, it has gained popularity over the past decade and has become an easy and recognized tool to evaluate scientific performance [16, 49]. We retrieved the h-indexes of all RN members through *Harzing’s Publish or Perish*, a free software program that calculates the score based on a scholar’s available publications in Google Scholar [50]. Other databases also offer the possibility of calculating the h-index, and scores can vary considerably from one database to another (e.g. Scopus, Web of Science). Studies have shown that, overall, none of these databases stands out as consistently more reliable than the others, and each has different strengths and weaknesses [51, 52]. Considering this relative equality, we opted for Google Scholar because it usually covers a higher proportion of non-English journals, which was relevant for many of the RN’s researchers [53]. The h-index calculation largely favours scholars with longer careers, since they generally have more publications [49]. To reduce this bias, we calculated the h-index of each researcher over a five-year period only (2008–2012).

Despite its growing popularity and widespread use, the h-index cannot replace all other indicators of scientific performance, nor does it solve the difficulty of measuring performance. It gives only a limited indication of a researcher’s productivity. For example, a researcher who published 30 papers of which only six had at least six citations would have the same h-index (h = 6) as a researcher who published just six papers that were cited at least six times each. The h-index is also a performance measure that is exclusively based on publications. This is a limitation, as the number and visibility of publications are to a large extent influenced by social factors such as professional reputation and notoriety [54] [55]. As Ioannidis graphically puts it, "It is sometimes difficult to tell whether a superb CV with a lengthy publication list reflects hard work and brilliant leadership or the composite product of dexterous power game networking, gift authorship and excellence in the slave trade of younger researchers” [56].

Even though this process does not solve the inherent complexity of measuring performance, it is advisable to combine different indicators of performance [49]. We thus computed a CV-based customized productivity index (CPI) that takes into account a larger sample of research output. We compiled from each member’s CCV: 1) the total amount of funding received in ongoing grants; 2) the number of ongoing student supervisions; 3) the number of published articles in peer-reviewed journals over the past five years (2008–2012); and 4) the number of other publications in the same period (e.g. books and book chapters, research reports, articles in non peer-reviewed journals). We did not include communications in this productivity indicator because they involve much less work than do publications and grant submissions. Also, we decided to count the total amount received in grants rather than the number of grants received, based on the assumption that one large grant involves a higher workload than a few small grants. All four categories of research activities were standardized and weighted according to the following scale: grants = 2, peer-reviewed publications = 2, supervised students = 1, other publications = 1. The weighting reflects the values that peer review committees in the RN's field attribute to these different kinds of achievements. The final CPI score is the sum of these four standardized weighted scores.

### Correlation analysis

To measure the association between structural position and performance we computed correlation scores between the measure of structural position (BC) and the two performance indexes. We believe BC is the most relevant structural measure to assess this association, for two reasons. The first is derived from graph theory, which posits that network connectivity depends primarily on shortest paths. This implies that if researcher A is on the shortest path between researchers B and C, then A will have a more important role in information transmission between B and C than will researcher D, who also connects B and C, but through a longer, more convoluted path. The second reason is anchored in sociological applications of Granovetter’s [1] and Burt’s [2] theories on weak ties and structural holes. The structural impact of researchers’ developing new but redundant ties with close colleagues with whom they regularly collaborate is likely very limited. Such ties are unlikely to bring new information or new opportunities. In the same way, even a non-redundant new tie to a colleague who belongs to an existing cluster of people with whom one regularly collaborates has a limited impact on information and opportunities. On the other hand, developing a new collaboration with a researcher belonging to a cluster of people with whom no or few ties currently exist is likely to provide new insights, new ideas, new projects and, more generally, opportunities for performance improvement.

To measure the correlations between BC and the performance indexes, we first used Pearson’s r coefficient. However, as this coefficient is sensitive to extreme values, we also used Spearman’s Rho. Simple linear regression coefficients with 95% confidence intervals were calculated to measure the strength of the association between the variables of centrality and each of the two performance variables. These analyses were then repeated after eliminating individuals with extreme values (mean + 3 standard deviations). The correlation analyses were performed with SPSS 22 software.

## Results

Before presenting the results, a short discussion of the nature of the network we sampled may be useful. Earlier we suggested that it would be theoretically possible to map out the entire ‘scientific community’ of all individuals involved in the human scientific endeavour and all their collaborative ties at one given time. Against the backdrop of this imagined underlying worldwide network, what we have done here is equivalent to extracting from that complete network a sample made up of all RN members and all their collaborators and connections. This means that, relative to the complete network, our sample includes all the connections of all RN members, and all ties that do not involve at least one RN member are missing, or ‘broken’. As such, we did a form of RN-centric sampling. As stated earlier, we believe that, both conceptually and methodologically, there is no way to conduct SNA analysis of scientific collaborations without some sampling bias. We will return to this issue in the discussion, but it is important to understand that the network analyzed here is a very specific sample.

Through the data extracted from the CCVs of the 73 members of the RN network, we identified 2,360 researchers (including 2,287 non-RN members) and 7,018 collaboration ties among them. The number of collaborations per RN member researcher (degree of the node) ranged from 1 to 365, for an average of 115 collaborations established with an average of 52 different persons (minimum 1, maximum 143). The number of intra-RN collaborations (collaborations between RN member researchers) ranged from 1 to 106, for an average of 35 collaborations with an average of 5.4 different members (minimum 0, maximum 22).

### Structural position analysis

**Fig 1:**
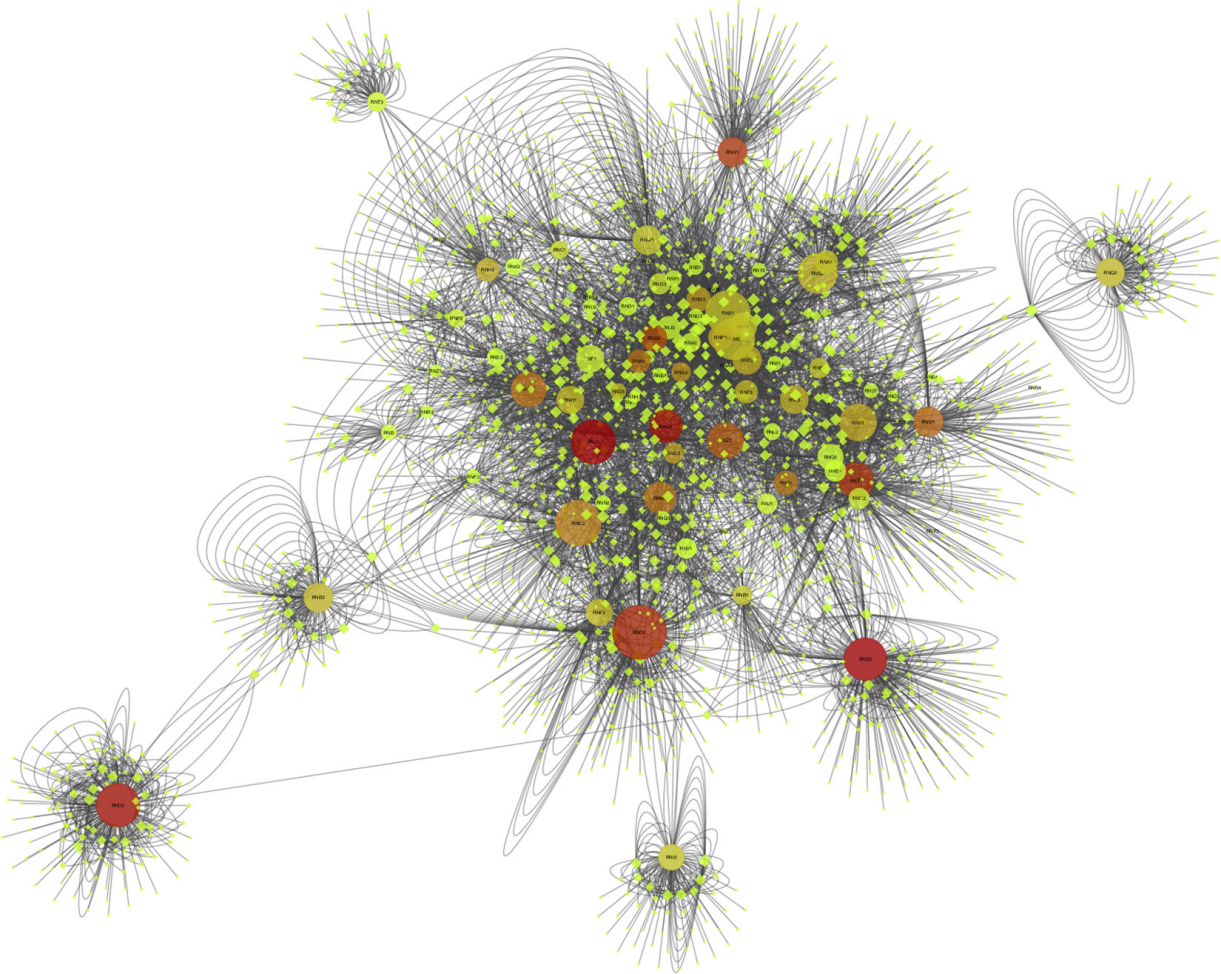
Collaboration network of RN members.

Visual analysis of the sociogram shows a network with a dense core of interconnections and a periphery constituted of RN members working mostly with non-RN members (Fig 1). Given the optimization algorithm used to draw the sociogram, highly interconnected researchers are positioned closer to the centre of the graph, while those with fewer connections or mostly connected to researchers with few connections are positioned at the periphery.

Visual analysis suggests that, in most cases, RN members with the highest number of collaborations (degree, plotted as node size) tend to have higher BC (reddish colour) as well. Correlation scores confirm this relation, as discussed below. However, beyond this general co-variation, the sociogram's visual analysis points to researchers with atypical positions or structural scores. For example, the researcher coded RNX1 has a high degree (284) with an average BC (0.039), which means he or she might have several redundant ties (a large number of collaborations established with a few collaborators). Conversely, the RN member RNN1 has fewer collaborations (163) but higher BC (0.084). This researcher thus occupies a central broker position between other researchers despite a lower number of collaborations.

It is also interesting to see that some highly productive members of the RN are at the periphery and others, such as RND2 (big reddish node on the left of the graph), barely have connections with the network core and have no connections with other RN members. By comparison, RNJ2 (big reddish node just left of and below the graph centre), whose structural metrics are similar to those of RND2, is very close to the centre of the RN network. In fact, visual analysis suggests no obvious link between distance to the core of the RN network and the two structural metrics (degree and BC) plotted on the graph. This is likely explained by the sampling strategy used.

### Correlation analysis

Table 1 provides basic information on structural position (centrality) and academic performance variables, while Table 2 provides the correlation scores and regression coefficients between BC and the two performance indicators.

**Table 1:** Descriptive analysis of centrality and performance variables for RN members (n = 73).

	Betweenness centrality (2012)	Degree (2012)	h-index (2008–2012)	Customized productivity index (2008–2012)
Mean	.036	115.23	5.66	0
Standard deviation	.028	9.614	4.404	4.283
Minimum	0	1	0	-4.913
Maximum	.118	365	21	18.449

**Table 2:** Correlation and simple linear regression coefficients between betweenness centrality and both performance measures (h-index and CPI).

Betweenness centrality (2012)		h-index (2008–2012)	Customized productivity index (2008–2012)
N = 73 (entire sample)	Pearson’s correlation	.684**	.687**
	Spearman’s Rho	.636**	.753**
	Linear regression coefficient (CI (95%))	24.171 (18.078; 30.265)	103.977 (77.979; 129.974)
N = 70 (sample after removal of 3 individuals with extreme productivity values)	Pearson’s correlation	.648**	.666**
	Spearman’s Rho	.592**	.725**
	Linear regression coefficient (CI (95%))	18.384 (13.156; 23.612)	73.514 (53.574; 93.454)

p <0.001.

As visual analysis suggested, degree and BC are significantly correlated (Pearson’s r: 0.8; Spearman’s Rho: 0.851). This is to be expected, as an increase in the number of connections (degree) is likely to increase the number of non-redundant paths and hence the likeliness of opening a new shortest path between some actors in the network. However, only non-redundant new collaborative connections will have this potential.

In the same way, the two performance metrics used, the h-index and CPI, are highly correlated with each other (Pearson’s r: 0.797; Spearman’s Rho: 0.716). This correlation was expected as well, since quantity of publications is a variable used in calculating both indicators. Although we believe the h-index and CPI measure two different dimensions of academic performance, the two metrics were expected to be correlated.

More interestingly, our data strongly support the hypothesis that researchers’ structural position is associated with performance measures. The two performance measures used here are highly correlated with BC (Pearson’s r: 0.684 and 0.687; Spearman’s Rho: 0.636 to 0.753; all p >0.001). After individuals with extreme values were removed, the coefficients of variation decreased slightly but remained within the same ranges. The simple linear regression coefficients also suggest a strong association between BC and the two performance measures: for each increase of 0.01 in BC, the h-index increased by 0.24 (β = 24.171, CI 95% (18.078; 30.265), p <0.001) and the CPI increased by 1.04 (β = 103.977, CI 95% (77.979; 129.974) p <0.001). These coefficients were lower when individuals with extreme values were not considered, but nevertheless remained highly significant.

For illustration purposes, we chose three RN researchers with different h-indexes (3, 8 and 19) and artificially added to each of them the same set of three randomly chosen new non-redundant ties with fellow RN members. This increased our selected researchers’ BC scores by 0.030, 0.043 and 0.024, respectively. Of course, researchers establish collaborations for a multitude of different reasons, such as personal affinity, shared research interests or professional aspirations, and the creation of new collaborations cannot be controlled in the way we did in this scenario. Nevertheless, it is interesting to see that, considering the regression coefficients obtained in our research, this would mean a theoretical impact on the selected researchers' h-indexes of, respectively, 0.73; 1.04 and 0.57. For the most junior researcher with the h-index of 3, this represents an impressive 24% increase (and 13% and 3% increases, respectively, for the other two).

## Conclusions

The objectives of this study were threefold: 1) to discuss the conceptualization of collaboration networks underpinning scientific endeavour; 2) to showcase a method of mapping those networks; and 3) to examine the association between researchers’ structural positions in such networks and their academic performance.

Both the methods we used to map the collaboration network or RN members and the underlying conceptualization of the scientific communities were significant, as we found a strong association between RN members’ structural position and two measures of their academic performance. The most interesting correlation identified was between BC and the h-index. One possible explanation for their co-variation could be that researchers occupying a bridging position in a network are more likely to have a higher publication performance than those connected mostly with the same small group of researchers in all their activities. If this is the case, it would imply, on a practical level, that if a researcher has an idea for a new paper or project, it would be more advantageous to find new collaborators and link with new teams than to work with the usual collaborators. This explanation of the statistical association is consistent with a few other similar studies [16, 17, 57]. Moreover, as discussed earlier, there are convincing sociological foundations for the idea that structural position influences social and market success [2, 58]. Our view is that collaborative relations form part of a researcher’s social capital, as they provide commodities, such as research ideas and financial resources, or opportunities to join other research projects and to exchange knowledge with experienced peers [59]. In turn, those commodities translate into researchers’ actual scientific performance, although only in part. This postulate tallies with the idea that BC is important, as a researcher in a bridging position will have greater access to such commodities than will one who maintains redundant ties with a closed cluster of individuals. Being in this bridging position has the advantage of giving access to a wider variety of knowledge and opportunities. In other words, researchers with a high level of social capital are regularly in bridging positions between groups and communities and are ultimately more likely to produce influential work.

A second finding of interest had to do with the role of the RN network. The significant difference in numbers of external and internal collaborations showed that the RN is only one source of collaboration among many others. The visual analysis results suggested that, even when sampling the network from an RN-centric perspective, the RN network was not a core explanatory factor for its members’ structural centrality. For example, some researchers positioned at the periphery of the graph and with very limited ties to the RN’s core displayed very high BC scores. This means that being part of the RN network is not sufficient in itself to improve performance. The recentness of this network’s creation may explain why some of the most bridging and highest-performing researchers are not necessarily at the core of the network and rely more on external collaborations. Other organizational characteristics of the network might also be at play, such as the fact that the network does not represent a significant funding source for its members and that participation in the activities of the centre is on a voluntary basis.

These findings bring us to the central questions of whether it would be possible to deliberately modify a researcher’s structural position and to what extent this could influence his or her scientific productivity. The strength of the association we found between structural metrics and performance scores exceeded what we anticipated. The idea that a researcher could gain up to one h-index point through new connections with three new colleagues could, in our view, definitely be of interest to researchers, university managers and grant funders. Indeed, funding agencies increasingly promote the creation of networks to support collaborative research, and the funding and sustainability of these institutional networks depend on their capacity to demonstrate network-level impact. As our study of the RN network demonstrated, these institutional structures do not fit squarely with the real—empirically underlying—collaboration networks uniting researchers. This opens up two avenues for funding agencies. One would be to target more funds to support collaborative infrastructures on the basis of some empirical demonstration of their ‘organic’ nature. The second, more in line with current funding rules in Quebec, is based on the expectation that funding collaborative infrastructures will drive practice and eventually influence researchers' behaviour. This second approach is based largely on the hypothesis that collaborative infrastructures are instruments able to modify research practice. Our results suggest that collaboration networks’ structures are indeed strongly associated with scientific productivity, but how deliberately modifiable they are remains unknown and constitutes an important avenue for future research.

One major challenge is the extreme methodological difficulty of attributing changes in the average member’s productivity to the research network’s existence or activities, because the actual structures of collaboration formed by researchers never overlap completely with institutional structures. Using SNA mapping to analyze the interconnectedness and structural positions of a research institution’s members may be helpful in advancing research on this question. Some authors have used SNA methods to enable ‘strategic management’ of networks, albeit in relation to different research topics [60, 61]. In Brazil, for example, Morel et al. [61] demonstrated how decision-makers used the mapping of medical science institutional networks to stimulate research on neglected diseases by strengthening local institutions that held strategic bridging positions in regional and international networks. With the method we developed, it would be possible to apply such an approach to research centre members. For example, we could hypothetically program an algorithm that would identify, for each researcher in the network, which collaboration ties could maximize BC and potentially have a significant impact on research productivity. In a research network such as the RN, our mapping could be used to offer incoming junior researchers mentorships with more senior researchers in ways that maximize the juniors' BC. Likewise, it would be possible to identify who should connect with whom to maximize the BC gain per new connection. But would the deliberate and instrumental creation of new links between researchers actually produce outcomes? The creation of successful and productive collaboration links depends, of course, on a host of factors, such as personal affinity, working styles and geographic or linguistic proximity [59, 62]. Furthermore, factors related to the research network itself, such as scientific leadership approaches, organizational structure and communication mechanisms, might also have an influence on the creation of deliberate linkages that can increase academic performance [62]. Finally, it is hard to predict with certainty that these ‘suggested’ collaborations will result in productivity increases, since, as stated earlier, researchers’ quantifiable performance is mediated by many of their social characteristics, such as their level of peer recognition, the reputation of their institution of affiliation, and their career stage, gender or nationality. These are questions that experimental interventions could help answer.

## Limitations and Future Research

This study has certain limitations. First, there might be other explanations for the strong association we found between BC and the performance measures. For example, it may be, conversely, that the most influential researchers attract a larger number of collaborators and are thus more likely to act as bridges between different groups of researchers. An elegant way to further assess the plausibility of our explanation would be to follow the same sample of researchers longitudinally over several years [63]. If structural position indeed influences performance, as we suggest, then the temporal pattern would show an increase in BC followed by a rise in performance. Longitudinal tracking of researchers’ careers taking into account the linkage and mentorship support they received would also provide interesting insights into the practical feasibility of managing scientific communities strategically. We also cannot exclude the possibility that other confounding factors might explain the association between BC and performance.

Second, even when compiling all the possible collaborations available in the CCV of a researcher, we still cannot capture certain forms of ties between researchers, such as informal relationships [17]. Lee and Bozeman [11] argue that the CV method consists of a basic counting of actual collaborations but does not provide an assessment of a researcher’s understanding of significant relations. Despite its shortcomings, the data collection method based on CVs has the advantage of being an accessible, unchanging and verifiable source of information while capturing a wider range of collaboration ties than co-authorship networks. Moreover, for evaluation purposes, it is the most useful and straightforward method [23].

This study could also benefit from being replicated with other data sets to take into account the variety of factors that influence researchers’ ability to build collaborations that improve social capital and scientific performance, such as motives for collaboration [11], geographical location [25, 26], number of international collaborators [13], individual capacities, and funding resources [17, 64, 65]. For example, Lee and Bozeman argue that although their research—measuring the effects of the number of collaborations on the number of journal publications produced by a researcher—shows a strong correlation between the number of collaborators and publications production, other elements also come into play, such as academic discipline, motives for seeking collaborations and seniority [11].

Finally, the RN-centric sampling provides only a partial view of the larger underlying scientific community network. Considering the strength of the correlations found in the RN network study, it is plausible that the whole-network structural position would have an even stronger effect on academic performance than what was measured here.

As mentioned, if indeed structural position explains a significant proportion of productivity and influence, then structural analysis could rapidly become a useful tool for research centres, universities and funding agencies. The data collection method based on CVs could be easily used to map large research networks, especially since there is now a proliferation of large-scale CV databases imposed by funding agencies, such as the Canadian Common CV. These databases render the automatic computation of researcher-based centrality measures technically feasible on a national scale. We believe it is only a question of time, and likely not a long one, before such evaluation practice develops. If this information is connected to what companies such as Google are doing in terms of computing h-factors on a large scale, then new horizons are opening up to assess empirically how scientific communities are structured and what impact structural position has on individuals’ and subgroups’ productivity. Now is the time for academics to understand the likely effects of such a trend and to enter into a collective discussion about the most socially desirable indicators of scientific performance.

## Supporting Information

S1 File Kernel Density Estimations (KDE) of the four variables. Figure 1: Betweenness Centrality, Figure 2: Degree, Figure 3: H-index, Figure 4: Customized Productivity Index.
(PDF)
