# Supplementary material for: The Impact of a Researcher’s Structural Position on Scientific Performance: An Empirical Analysis
Source: PLoS One. 2016 Aug 31;11(8):e0161281. doi: 10.1371/journal.pone.0161281 (PMC5006965; doi:10.1371/journal.pone.0161281)

## Kernel Density Estimations (KDE) of the four variables:

## Betweenness Centrality, Degree, H-index, Customized Productivity Index

### Figure 1: Betweenness Centrality: Probability Density Function Estimation

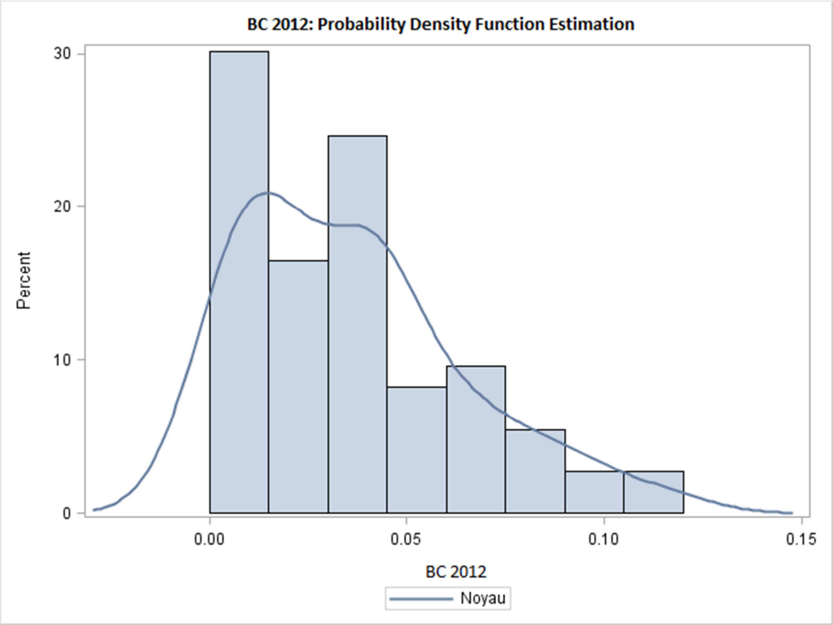


### Figure 2: Degree: Probability Density Function Estimation

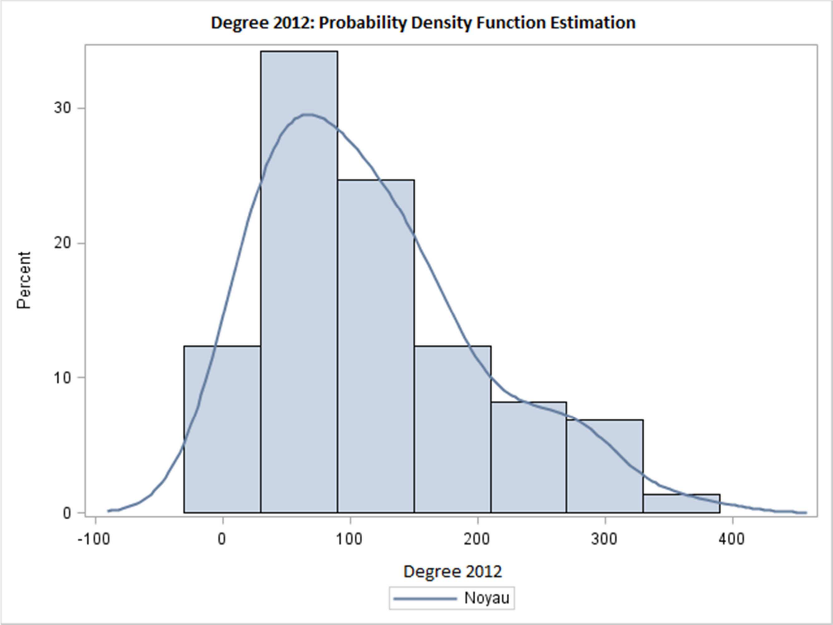

**Figure 3: H-index: Probability Density Function Estimation**

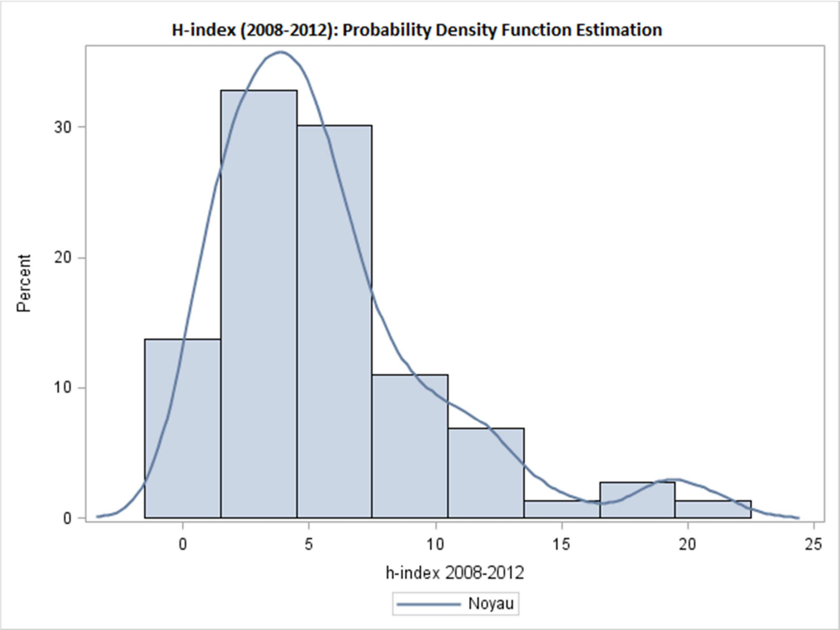


**Figure 4: Customized Productivity Index: Probability Density Function Estimation**

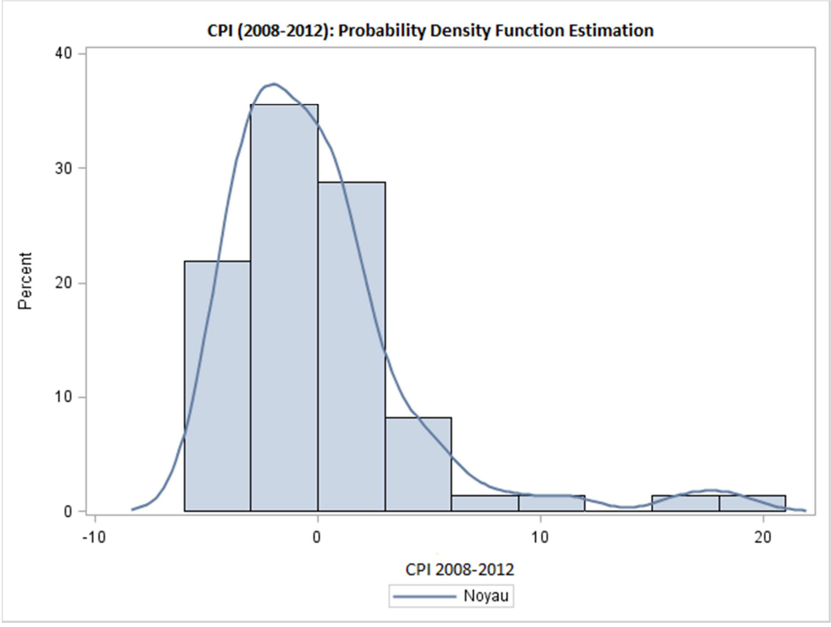

Supplement: S1 File — Figure 1: Betweenness Centrality, Figure 2: Degree, Figure 3: H-index, Figure 4: Customized Productivity Index. (PDF) [file pone.0161281.s001.pdf]
